# Evaluating the Benefits of Aphasia Intervention Delivered in Virtual Reality: Results of a Quasi-Randomised Study

**DOI:** 10.1371/journal.pone.0160381

**Published:** 2016-08-12

**Authors:** Jane Marshall, Tracey Booth, Niamh Devane, Julia Galliers, Helen Greenwood, Katerina Hilari, Richard Talbot, Stephanie Wilson, Celia Woolf

**Affiliations:** 1 Division of Language and Communication Science, City University London, London, United Kingdom; 2 Centre for Human Computer Interaction Design, City University London, London, United Kingdom; University College London, UNITED KINGDOM

## Abstract

**Introduction:**

This study evaluated an intervention for people with aphasia delivered in a novel virtual reality platform called EVA Park. EVA Park contains a number of functional and fantastic locations and allows for interactive communication between multiple users. Twenty people with aphasia had 5 weeks’ intervention, during which they received daily language stimulation sessions in EVA Park from a support worker. The study employed a quasi randomised design, which compared a group that received immediate intervention with a waitlist control group. Outcome measures explored the effects of intervention on communication and language skills, communicative confidence and feelings of social isolation. Compliance with the intervention was also explored through attrition and usage data.

**Results:**

There was excellent compliance with the intervention, with no participants lost to follow up and most (18/20) receiving at least 88% of the intended treatment dose. Intervention brought about significant gains on a measure of functional communication. Gains were achieved by both groups of participants, once intervention was received, and were well maintained. Changes on the measures of communicative confidence and feelings of social isolation were not achieved. Results are discussed with reference to previous aphasia therapy findings.

## Introduction

Aphasia is the acquired loss of language following brain damage, most commonly caused by stroke. A recent analysis of pooled trial data concluded that 45% of stroke survivors acquire aphasia, with 24% having persistent symptoms [1].

Aphasia varies across individuals, both in terms of type and severity [2]. For some people, speech is obliterated or reduced to a few words, while others retain fluent but errorful speech. Difficulties with reading, writing and the understanding of speech are also common, although again with varying presentations. Aphasia has profound consequences for a person’s quality of life [3, 4, 5]. Effects on personal and social relationships are particularly devastating, with loss of friends commonly reported [6].

The problems of aphasia are responsive to treatment. The latest Cochrane review on speech and language therapy following stroke concluded that therapy can enhance functional communication, reading, writing and expressive language [7]. Most comparisons across treatments were inconclusive. High intensity or long term therapy was associated with benefit, although such regimes were also associated with high rates of drop out.

The heterogeneity of aphasia makes it unlikely that any single intervention can meet all needs. Rather, diverse treatment approaches are called for [8]. In recent years this diversity has been augmented by applications of digital technology [9]. Computer therapy tools can deliver personally tailored exercises that benefit a range of language skills, including word retrieval [10, 11], verb production [12] sentence building [13,14], and speech comprehension [15]. Technology can also help to compensate for language impairments, for example by using text to speech software [16, 17, 18]. The response of users to computer based treatments has been positive [19, 20, 21] and tools have been made accessible even to people with severe aphasia [22].

Further benefits of technology relate to treatment delivery. Remote video conferencing technologies can be used both to assess [23, 24] and treat [25] people with aphasia, and so cater for individuals who cannot travel to clinic. Self directed computer practice can also raise the treatment dose. For example, Palmer et al [11] found that participants undertook an average of 25 hours of language practice on the computer, while receiving just under 5 hours of face to face contact with a Speech and Language Therapist (SLT).

Although aphasia therapy has begun to embrace technology, much of the potential remains under-explored. Many applications to date involve digital versions of familiar therapy exercises. In effect, the user performs tasks on a screen that could be carried out with pencil and paper. Few studies have explored novel therapeutic opportunities arising from gaming technologies (although see [22]) or from social media.

An area that particularly merits further exploration is virtual reality. This involves a computer-generated simulation of an environment with which the user can interact. Virtual reality offers a number of potential benefits for aphasia therapy. It can deliver a playful, immersive experience that may raise motivation, and so encourage intensive language practice. Drop out might be similarly inhibited. Virtual reality may help to reduce feelings of embarrassment that can accompany real world communication failure, so encourage the practice of difficult communication exchanges. Related to this, generalisation of therapy skills, from the clinic to the real world, may be promoted. Virtual environments that enable people with aphasia to meet others may enhance social contact and reduce feelings of isolation.

Uses of virtual reality in healthcare are widespread, ranging from the treatment of physical impairments [26, 27] to pain management [28]. There have been several promising attempts to treat emotional and cognitive disorders, including posttraumatic stress disorder [29], social anxiety [30, 31] anxiety in autism [32] and paediatric disorders of attention [33].

A number of studies have explored applications of virtual reality for people with communication difficulties, and particularly communication anxiety. Brundage and Hancock [34] invited 10 adults who stutter to give speeches to a live audience and to two virtual audiences, seen though a head mounted display. One of the virtual audiences was particularly challenging; i.e. members were inattentive, with some falling asleep. Findings showed that the virtual conditions elicited the same speaking anxieties and very similar levels of stuttering as the live condition. Correlations were closest for the challenging and live audience. These findings, supported by the results of qualitative interviews, indicated that the virtual experience was perceived as highly authentic. The authors concluded, therefore, that virtual reality could be used to remediate the communication anxieties and avoidance behaviours that are often associated with stuttering.

Speaking phobias have been addressed with virtual reality [35, 36]. Wallach et al [37] compared Cognitive Behavioural Therapy (CBT) with a virtual reality based CBT in the treatment of public speaking anxiety. The virtual reality component of therapy involved speaking assignments in front of a virtual audience, projected on a headset. Results showed that both treatment groups achieved significantly better outcomes than a waitlist control group. Scores between the treatment groups did not differ, although there were fewer drop outs in the virtual condition. Encouragingly, treatment gains were maintained for both groups at one year follow up [38].

Turning to stroke, several studies have explored uses of virtual reality in physiotherapy and/or occupational therapy [39]. Benefits have been described for upper and lower limb function, gait, and for everyday activities such as using a hammer or drinking from a cup [40, 41, 42, 43]. Two meta analyses, comparing virtual reality treatments with conventional therapy, showed that results favoured the former [40, 41]. It seems, therefore, that virtual reality techniques are acceptable to stroke survivors and that they can augment physical recovery. Effects presumably derive because participants are performing movements in the simulated environment that mirror those used in real life. Intriguingly there is also evidence that simply viewing avatar movements excites cortical regions involved in motor preparation [44], suggesting that virtual reality can be used to stimulate brain regions involved in motor recovery, even if movements are not specifically practised.

To date, very few treatments for aphasia have deployed virtual reality. Stark and colleagues developed a virtual house to promote language practice [45]. Two other programmes, Orla and AphasiaScripts [46, 47], make use of a virtual speech and language therapist. For example AphasiaScripts aims to improve speech production by practising scripted dialogues with the help of the avatar therapist. A series of case and small group studies has shown that AphasiaScripts is effective in teaching target dialogues and reducing speech errors, with good maintenance of therapy gains [48, 49]. Participant views are also positive [21].

None of the above aphasia applications allow for multi user language practice in a virtual world. This paper describes a new virtual communication environment for people with aphasia, called EVA Park, which does provide this opportunity. First we introduce EVA Park. We then present the results of a pilot intervention, in which twenty people with aphasia had 5 weeks access to EVA Park. We explore compliance with intervention and use of the platform. The effects of intervention on participants’ communication, confidence, and feelings of social isolation are also appraised. The study addressed the following research questions:

- Will participants with aphasia comply with a regime of daily virtual intervention (5 sessions per week), as assessed by attrition and usage data?
- Will 5 weeks intervention in EVA Park improve performance on a measure of functional, everyday communication (Communication Activities of Daily Living, CADL-2, [50]); will there be additional gains on measures of verbal fluency, word finding in conversation and narrative?
- Will intervention enhance feelings of communicative confidence, as assessed by the Communication Confidence Rating Scale for Aphasia [51]?
- Will intervention reduce feelings of social isolation, as assessed by the Friendship Scale [52]?

Good compliance with intervention was predicted, as EVA Park was designed to be highly accessible to its user community. Effects of intervention were more difficult to predict as this was the first evaluation of multi user virtual reality therapy with people who have aphasia. The CADL-2 was predicted to improve as this assesses skills that were similar to those practised in EVA Park. Gains on the other aspects of language were not strongly predicted, but might occur as a result of the speech and conversation activities made available in EVA Park. We hypothesised that communicative confidence would improve, given that virtual applications have been successful in treating communication anxieties in other groups. We anticipated possible benefits with respect to feelings of isolation, given that participants were meeting others in EVA Park.

## Methods

The trial protocol and TREND check list are available as supporting information (S1 File and S1 Table). The trial was not registered prior to recruitment as the target sample size was low and this was not required by the ethics committee. The authors confirm that any ongoing and related trials for this intervention will be registered.

### Introduction to EVA Park

EVA Park is an online virtual island developed for the OpenSimulator platform. It was created in collaboration with people who have aphasia via a process of participatory design [53]. This process particularly ensured that EVA Park could be easily accessed and navigated by people with aphasia. For example, dependence on written menus was reduced to a minimum. EVA Park contains a number of simulated locations including houses, a cafe, a restaurant, a health centre, a hair salon, a tropical bar and a disco. There are green spaces, water features, wild life and elements of fantasy. For example, visitors to the narrow boat find a planetarium inside it, and those who dive into the lake discover a mermaid and a giant turtle. The environment is interactive. So, if users click on the turtle they are taken for a ride.

EVA Park can be populated by several people at the same time, each of whom is represented by a personalised avatar. Users communicate via speech in real time, using a head set and microphone. They can also optionally type. Avatars move round the island by walking, running or flying. Users navigate their avatars via a simple 6 button key pad, which sets the direction of movement, and via a mouse which can click on specific locations.

### The Intervention Study

#### Ethical clearance

The study gained clearance from the ethics committee of the School of Health Science, City University London (date of approval: 21.12.2012; Reference: LCS/PR/Staff/12-13/05). All participants gave written informed consent. Information and consent materials were designed to be accessible to people with aphasia.

#### Participants

**Fig 1:**
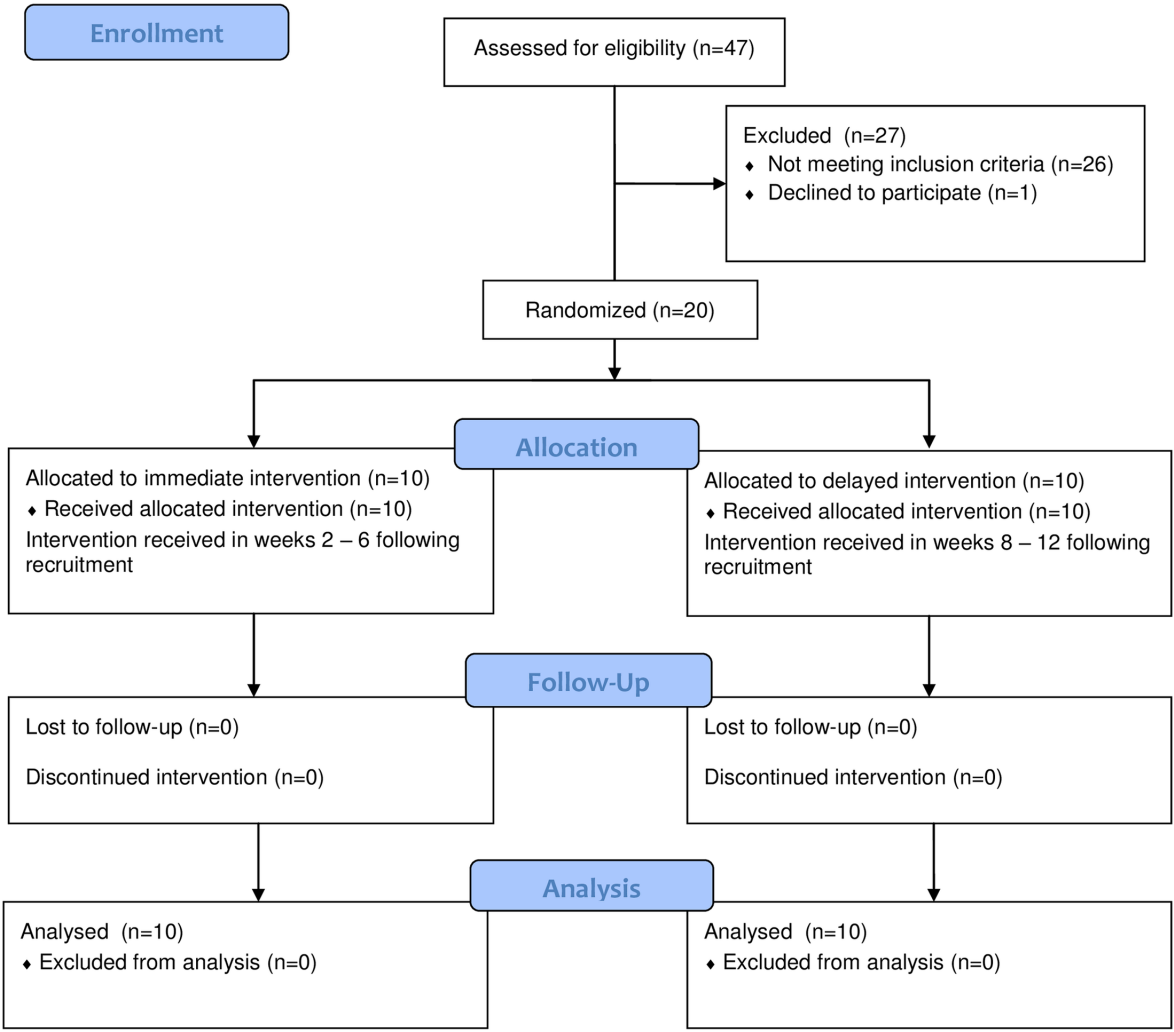
Consort Flow Diagram.

Twenty people with aphasia completed the intervention study (see Fig 1). They met the following criteria: all had a diagnosis of aphasia following a stroke that occurred at least 4 months prior to the study; they were fluent users of English prior to their stroke (self report); they had no uncorrected visual impairment (self report) and no hearing loss above 40Db (screened via pure tone audiometry); all had some spoken output (scoring at least 20% correct on the picture naming subtest of the Comprehensive Aphasia Test, CAT [54]); none had severe impairments of speech comprehension (scoring above 70% correct on the CAT test of Spoken Word to Picture Matching; and above chance on the CAT test of Sentence to Picture Matching). Participants needed to demonstrate impaired functional communication and wish to address this via EVA Park intervention (established via observation and discussion during screening). Participants were asked about their prior computer use, but responses did not determine inclusion in the study.

Participants were recruited from community groups for people with stroke and aphasia across London. They were referred by the group leaders or self-referred. Data were collected in participants’ homes or at City University London. Participants were not paid for their involvement and received no other incentives. Data collection, from first participant recruitment to final follow up, ran from 3^rd^ September 2013 to 29^th^ April 2015.

#### Content of Intervention

Each participant had 5 weeks access to EVA Park, in which they received supported language stimulation. They had daily sessions with a support worker (25 sessions in total), each lasting about one hour, supplemented by unlimited independent access. Thus participants could visit EVA Park at any time when they might meet and communicate with other participants. Once a week all participants and their support workers met for an hour long group discussion. Participants accessed EVA Park from their home, using laptops loaned from the University. With the exception of the initial technological set up, all intervention took place in EVA Park; i.e. support workers did not visit participants at home, rather they met with them at a specified time and place in EVA Park. There were four intervention cohorts, each involving five participants.

Each participant was paired with a support worker who helped them to set functional communication goals, and address those goals through communication activities and practice. Most support workers were qualified SLTs, although two were experienced stroke group volunteers (the volunteers worked with both intervention groups; see Design below). Support workers were provided with four hours of training prior to the intervention, which covered access to EVA Park, navigation, interaction skills, and communication activities. They also received weekly supervision from SLT researchers during intervention.

The content of intervention was largely driven by the personal goals set by each participant. All participants set at least three goals, and these were various. Some targeted specific aspects of language such as: asking questions, initiating conversation, and improving word finding. Other goals were more context bound, such as ordering food in a restaurant, making a doctor’s appointment, and enquiring about swimming classes. Goals were motivated by experiences in participants’ lives. For example, one participant was concerned about crime levels in her neighbourhood, so wanted to practise reporting a crime to the police. Participants and their support workers planned activities together to address the goals. For example, one participant, who aimed to improve word finding, decided to find and name all the animals in EVA Park; another worked on questions that he could ask during the group discussions. Role plays were common; for example participants practised requesting a hair cut, ordering food, making purchases and arranging appointments. One participant, who wanted to improve his ability to make points in an argument, held a meeting with other participants to discuss the benefits of building a sports centre in EVA Park. In all cases, these role plays were located in appropriate settings in EVA Park, with support workers playing various roles.

In addition to the goal directed activities, EVA Park was a platform for conversation, and sessions were often spent simply talking. Many features of EVA Park were designed to stimulate conversation. For example, the town centre contained a news board, which played topical videos when clicked. We also ran an election narrative during each intervention period in which four fictional candidates were standing for the position of Mayor. Candidates released manifestos, which could be discussed, and were the subject of (often scurrilous) gossip. Group discussions were a further opportunity for conversation, with topics ranging from the news, the royal family, music and celebrities.

### Design

The study employed a quasi randomised controlled design, which compared a group that received immediate EVA Park intervention with a waitlist control group. Participants were recruited in 4 cohorts. Two cohorts (1 & 4) were randomly allocated to the immediate group and two (2 & 3) to the waitlist control group. Assignment of the cohorts was determined at the outset of the study, before any recruitment took place. Participants were assigned to the cohorts in order of recruitment; i.e. the first five recruits were assigned to cohort one, the next five to cohort two and so on. Testing occurred at three time points following recruitment: week 1, week 7 and week 13. Participants in the immediate group received EVA Park intervention between week 2 and week 6, and no further intervention between week 7 and week 13. Participants in the waitlist control group received no intervention between week 1 and week 7; but received EVA Park intervention between week 8 and week 12 (see Fig 1).

#### Measures

All measures were administered at each time point.

Functional communication was assessed with the Communication Activities of Daily Living (CADL-2) test. This is a standardised assessment of everyday language for people with aphasia, which is based on specific scenarios, such as going to the doctor. It has demonstrated good inter-rater and test-retest reliability [50]. It has been widely used in intervention research [7] and has shown sensitivity to therapy induced change [55].

A verbal fluency task assessed word production. Participants were given one minute to name as many items as possible within a given category. Ten categories were tested. Five of these reflected the content of EVA Park (health centre, restaurant, park, kitchen, hair salon) and five did not (supermarket, airport, school, sports stadium, cinema). Participants were scored for the number of items named in each category, excluding repetitions, out of category responses and phonological errors. A total score, across the ten categories, was then derived.

Word finding in conversation was assessed using the POWERS procedure [56]. This has demonstrated good inter-rater reliability and sensitivity to therapy induced change [57, 58]. At each time point, participants were paired with a novel partner and asked to converse on a topic of their choosing. The partners were students of speech and language therapy who had received 2 hours training on conversation skills and were provided with standard instructions. The interaction was filmed and the middle 5 minutes were transcribed. Two indices were analysed from the participants’ output: the percentage of content words (nouns, verbs, adjectives and adverbs) against all words produced, and the number of content words per turn. Scoring was blind with respect to the assessment point and group assignment. Reliability of scoring was assessed by double coding one randomly selected conversation from five of the participants (8% of the data). Agreement was excellent (intraclass correlation coefficient = .99, p < .0001).

Narrative production was assessed by asking participants to re-tell the story of Cinderella at each time point. Data were elicited, transcribed and analysed using methods from the Quantitative Production Analysis [59]. This procedure has demonstrated good inter-rater and test/retest reliability, and sensitivity to therapy induced change [60]. Data from our study were transcribed and scored blind with respect to the assessment point and group assignment. A random sample of 15 narratives (25% of the data) were double coded to check reliability. Agreement was excellent (intraclass correlation coefficient, .99, p < .0001). The following indices were analysed: the number of narrative words per minute and the number of well formed sentences.

The Communication Confidence Rating Scale for Aphasia (CCRSA, [51, 61, 62] was used to assess communication confidence. This asks ten questions relating to different aspects of communication, such as ‘How confident do you feel about your ability to talk with people?’ Confidence is rated on a 0–100 scale, where 0 is ‘not confident’ and 100 is ‘very confident’. The measure is still under development, and sensitivity to change has not been established [61, 62]. It is the only published confidence measure designed to be used with people who have aphasia.

Feelings of social isolation were probed by the Friendship Scale [52]. This is a simple, 6 item questionnaire with good reliability and discriminant validity.

Qualitative data, comprising structured observations of participants using EVA Park and participant interviews were also collected. These will be presented elsewhere.

One further measure, the Social Network Analysis [63] was specified in our protocol. This requires respondents to name individuals in their social network. Data were subject to very high levels of individual variability over testing occasions, which seemed largely due to the naming difficulties of our participants. Results were not, therefore, informative about participants’ social contacts and were not analysed.

Measures were administered by the speech and language therapy researchers (ND, HG and RT), who were not blinded to time point or group allocation. As outlined above, it was possible to conduct blind scoring of some of the measures. Participants were not blind to their condition, i.e. they knew whether or not they had received intervention at the time of testing.

#### Analyses

T test or Mann Whitney comparisons probed for baseline differences between the groups.

For the outcome measures two ANOVA analyses were planned, following checking of the data to ensure that ANOVA assumptions (normality, homogeneity, sphericity) were met. The first was a mixed between-within subjects ANOVA (henceforth ‘mixed ANOVA’), with the within variable of time (two levels: week 1 and week 7) and the between variable of group (two levels: immediate and waitlist control). This compared the groups at two time points, between which the immediate group had received intervention and the waitlist control group had not. Thus, crucially, any treatment effect was signalled by a group x time interaction. The second analyses employed one factor, within group ANOVAs, comparing results at the three time points: week 1, week 7 and week 13. These examined change over time for each group separately. Where there was a significant main effect, or trend (p = .06), planned comparisons between each time point were conducted. Here treatment effects were signalled by a main effect of time, together with relevant planned comparisons. So, for the immediately treated group a treatment effect was indicated by a significant improvement between weeks 1 and 7, with maintenance of gain indicated by a significant improvement between weeks 1 and 13. For the waitlist control group, a treatment effect was indicated by a significant improvement between weeks 7 and 13. If data were not normally distributed, as assessed by the Shapiro Wilk test, non-parametric analyses were applied. These consisted of the Friedman Test with post hoc Wilkinson Signed Ranks tests (with Bonferroni adjustments).

A follow up analysis explored whether the gain on each outcome measure correlated with the amount of time that each participant spent in EVA Park. Where therapy induced change had been demonstrated on a measure, a follow up analysis of covariance examined pre to post therapy change with the time logged in EVA Park as a covariate.

All statistical analyses were conducted on IBM SPSS Statistics 22 software.

## Results

### Participant data (Table 1)

**Table 1:** Participant Data.

	Immediate Group	Waitlist Control Group	Total
Gender	4 women, 6 men	5 women, 5 men	9 women, 11 men
Age mean (s.d.)	59.00 (13.61)	56.6 (9.73)	57.8 (11.58)
Months post Stroke: Mean (s.d.)	70.10 (68.91)	54.1 (34.46)	62.10 (53.56)
Picture naming: Mean % correct (s.d)	80.37 (12.32)	69.74 (19.39)	75.05 (16.73)
Spoken word to picture matching test: Mean % correct (s.d.)	90.49 (6.89)	88.97 (9.15)	89.73 (7.92)
Spoken sentence to picture matching test: Mean % correct (s.d.)	82.81 (9.49)	70.56 (17.20)	76.68 (14.91)
Prior computer use: Mean number of computer uses (s.d.)	2.6 (1.58)	3.1 (1.45)	2.8 (1.50)

T test comparisons for age and screening measures found no significant baseline differences between the groups. The comparison for the sentence to picture matching test approached significance (p = .064). Data for time post stroke and picture naming were not normally distributed (Shapiro-Wilk Test p < .001 and p = .01 respectively), so were examined using the Mann Whitney test. Results were not significant.

Prior computer use was determined by asking each participant whether or not they had made use of seven different computer applications in the last month (email, skype, online shopping, Facebook/Twitter, computer games, computerised speech and language therapy exercises and accessing information on the internet). Only two participants indicated no computer use; all others were using at least one application. Data were not available for one participant. A t test comparison confirmed that there was no significant difference in computer use across the groups.

### Compliance with intervention

As illustrated by Fig 1, there was no attrition after randomisation and no participants lost to follow up. Ten participants completed all 25 scheduled sessions with their support worker and eighteen completed at least 22. The two remaining participants received 21 and 17 sessions. They both had ill health and family difficulties during the intervention period. One also struggled to use the platform.

The amount of time spent logged into EVA Park by each participant was automatically recorded. The mean value was 40.85 hours. The range was very wide extending from just under 14 hours to just over 100 (median 34.89). The amount of time spent in EVA Park was not influenced by gender (U = 35, p = .28) or age (r_s_ = .173). It was, however, affected by prior computer use (r_s_ = .652; p = 0.002). Those with higher computer use scores spent the most time in EVA Park.

### Outcome measures

Results on the outcome measures are reported in Table 2. With the exception of the narrative scores, for all results N = 10 for both the immediate and waitlist control groups. Box plots illustrating the results on all outcome measures are provided in the supplementary materials (Figs A-H in S3 File).

**Table 2:** Mean scores (s.d.) on all outcome measures across the three time points.

	Week 1		Week 7		Week 13	
	Immediate group	Waitlist control group	Immediate group	Waitlist control group	Immediate group	Waitlist control group
CADL-2 stanine score	6.5 (1.51)	6.2 (1.39)	7.2 (1.55)	6.1 (1.19)	7.4 (1.35)	7.0 (1.41)
Verbal Fluency*	75.8 (31.42)	52.9 (20.89)	82.0 (38.20)	62.5 (20.98)	94.0 (40.87)	72.7 (26.93)
Conversation % content words	29.0 (6.39)	25.6 (9.84)	30.6 (6.19)	25.4 (9.41)	30.6 (10.64)	24.8 (8.57)
Conversation content words/turn	3.1 (2.06)	2.6 (1.89)	3.1 (1.76)	2.3 (1.75)	2.6 (1.29)	2.7 (2.60)
Narrative words per minute	40.9 (20.75)	31.4 (25.18)	50.0 (29.98)	34.8 (19.32)	53.2 (28.38)	35.4 (21.95)~
Narrative sentences#	13.2 (6.76)	11.0 (6.34)	14.9 (6.69)	13.2 (6.79)	15.5 (7.26)	13.3 (8.65)~
CCRSA	29.9 (3.21)	22.5 (5.29)	32.2 (5.75)	26.4 (6.04)	33.8 (4.92)	29.2 (6.03)
Friendship Scale	17.3 (4.35)	15.5 (5.74)	19.2 (4.64)	16.6 (5.78)	18.2 (4.73)	17.3 (4.57)

* Mean number of items named over the 10 categories

^#^ Mean number of well formed sentences

^~^ N = 9; one participant declined the narrative assessment at week 13.

CADL-2: In the mixed ANOVA there was no main effect of time or group. However, there was a significant interaction (F (1,18) = 5.236, p = .034, η_p_^2^ = .225). Thus the immediately treated group improved between week 1 and week 7, whereas the waitlist control group did not. One factor analyses examined change over the three time points for each group. For the immediate group there was a significant main effect of time (F (2, 18) = 8.26, p < .003, η_p_^2^ = .48). Pairwise comparisons were significant for week 1 vs week 7 (p = .001) and for week 1 vs week 13 (p = .004) but not for week 7 vs 13 (p = .51). This analysis confirmed that the immediately treated group improved following intervention, and that the gain was maintained at week 13. Data at 13 weeks for the waitlist control group were not normally distributed (note that these data were not included in the mixed ANOVA). This group’s data were therefore analysed with the Friedman Test, with a highly significant result (Friedman χ^2^ = 10.14, p = .006). Post hoc comparisons, with alpha set at .016, were significant for week 7 vs week 13 (p = .014) and for week 1 vs week 13 (p = .011), but not for week 1 vs week 7 (p = .74). Thus the waitlist control group demonstrated a stable baseline, but improved on the CADL-2 once intervention was received.

Verbal fluency: The mixed ANOVA produced a main effect of time (F (1,18) = 6.54, p = .02, η_p_^2^ = .266), but no effect of group and no interaction. So, participants improved between week 1 and week 7 on this measure, but both groups improved equally. The one factor ANOVA produced a main effect of time for the immediate group (F (2,18) = 6.10, p = .009, η_p_^2^ = .404). Pairwise comparisons were significant for week 1 vs week 13 and for week 7 vs week 13 (both p < .05). There was also a main effect of time for the waitlist control group (F (2,18) = 11.28, p = .001, η_p_^2^ = .556). Pairwise comparisons were significant for week 1 vs week 7 (p = .025) and for week 1 vs week 13 (p < .001). These analyses confirmed that total naming scores increased over time, but gains were not tied to intervention.

The fluency task included 5 categories relating to EVA Park and 5 that were unrelated (see Table 3). Scores for these categories are broken down in Table 3. This shows that both groups improved on the EVA Park categories more than on the non EVA Park categories, particularly in the periods flanking intervention. However, this trend was not significant (p = .298, η_p_^2^ = .057).

**Table 3:** Mean verbal fluency scores (S.D.) on the EVA Park and non EVA Park categories over the three time points.

	Week 1		Week 7		Week 13	
	EVA Park Categories	Non EVA Park categories	EVA Park Categories	Non EVA Park Categories	EVA Park Categories	Non EVA Park Categories
Immediate Group	39.5 (16.91)	36.3 (15.78)	43.4 (21.02)	38.6 (17.58)	49.1 (20.31)	44.9 (21.52)
Waitlist control group	27.4 (12.95)	25.5 (8.59)	32.5 (13.02)	30.0 (10.14)	38.5 (14.34)	34.2 (13.59)

Word finding in conversation: The percentage of content words against all speech units was analysed through mixed and one factor ANOVAs, with no significant results. Data for the number of content words per turn were not normally distributed, so were analysed with separate Friedman Tests (for the immediate group and waitlist controls). There were no significant findings.

Narrative: The ANOVA analyses for both the number of narrative words produced per minute and the number of well formed sentences produced no significant findings.

Communication confidence: The mixed ANOVA produced a main effect of time (F (1, 18) = 7.50, p = .013, η_p_^2^ = .294) but no interaction. Thus change on the measure was not confined to participants who had received intervention. This analysis produced a main effect of group (F (1, 18) = 10.583, p = .004, η_p_^2^ = .37), with the immediate group scoring more highly than the waitlist control group. The one factor ANOVA approached significance for the immediate group (F (2, 18) = 3.31, p = .06, η_p_^2^ = .269). Only one pairwise comparison was significant for this group: week 1 vs week 13 (p = .034). There was a significant main effect for the waitlist control group (F (2,18) = 8.91, p = .002, η_p_^2^ = .497). Pairwise comparisons were significant for week 1 vs week 7 (p = .016) and week 1 vs week 13 (p = .007) but not for week 7 vs week 13.

Friendship Scale: There were no significant effects in the mixed or one factor ANOVAs.

### Impact of usage on outcome

The final analyses explored whether the amount of time spent logged into EVA Park influenced scores on the outcome measures. This was investigated initially by correlating the individual log-in times with the gains made on each measure. Gains were calculated by subtracting week 1 scores from week 7 scores for the immediately treated group and week 7 scores from week 13 scores for the waitlist control group. Values are reported in Table 4; N = 20 for all correlations except the narrative scores, where N = 19. There were two significant values. The percentage of content words in conversation showed a negative correlation and communicative confidence (CCRSA) showed a positive correlation.

**Table 4:** Correlations between time logged into EVA Park and gain scores on all outcome measures.

	CADL-2	Verbal Fluency	Conv* % content words	Conv* content words/turn	Narrative words per minute	Narrative sentences	CCRSA	Friendship Scale
r_s_	.252	.419	-.639	-.236	.159	.136	.446	.131
Sig	.28	.07	.002	.32	.52	.58	.048	.58

Conv* = Conversation.

Results for the CADL-2 scores, which had demonstrated a therapy effect, were subject to further analysis. Following a preliminary check of linearity, a repeated measures ANCOVA was run. This compared pre and post therapy scores on CADL-2 across the whole sample (N = 20) with time logged into EVA Park as the covariate. This showed that log time was not significant (F = .672, p = .42, η_p_^2^ = .036).

## Discussion

This study trialled a novel intervention for people with aphasia delivered through a virtual reality platform called EVA Park. It employed a quasi randomised controlled design, which compared the results of ten people who received immediate intervention with ten people who formed a waitlist control group. It examined compliance with intervention, and effects on a range of outcome measures, with predicted benefits for functional communication, communication confidence and feelings of social connectedness.

Compliance with the intervention was excellent. All participants completed the study and 18 missed no more than three supported intervention sessions. Reasons for missed sessions were ill health and adverse events within the family. The individual who missed the most sessions also experienced some difficulties in using the platform. For example, he struggled to log in and to navigate between locations in EVA Park. He was the only participant who displayed such difficulties.

Further evidence of compliance was provided by the automatic log in data showing that participants spent an average of 40.8 hours in EVA Park. As each person received no more than 25 hours of supported practice this indicates a high level of independent access. Indeed, fourteen of the participants logged at least 30 hours in EVA Park. Interestingly, usage was not affected by age or gender although it was by prior computer use.

The quality of participants’ independent use is difficult to judge, as it was unmonitored. It may have included communication practice with another participant, but this was by no means guaranteed. Some individuals commented that they enjoyed visiting different places in EVA Park, or finding attractive places to sit. Others said that independent use was boring because it was solitary.

The lack of attrition in this study compares very favourably with previous aphasia therapy research. A review of 57 RCTs involving 3002 participants, found that 17% of the pooled sample withdrew from intervention (N = 518) and 8% were lost to follow-up (N = 254) [7]. Although many withdrawals were for reasons unconnected to the therapy, such as illness or death, others were not. For example, 17 studies reported that participants self discharged, declined, withdrew from or could not tolerate therapy, and four reported losses due to difficulties with transport. The sample recruited in this study was relatively young (mean age <60 years). They were also, on average, over five years post stroke, and therefore medically stable. Some risks of attrition were therefore reduced. Nevertheless it is striking that only one person declined involvement (because they secured paid employment) and all completed intervention. Of course, the virtual nature of intervention meant that transport difficulties could not arise.

The positive attrition and usage data suggest that EVA Park is highly acceptable to people with aphasia. This may be a result of the design process, which ensured that the creation of EVA Park was informed by user opinion [53]. For example, our user informants stressed the need to make EVA Park a social and playful environment, and this seemed to be appreciated by the participants in this study. It is likely that the intervention format, and particularly the contribution of the support workers, was also crucial. The fact that our sample was, in most cases, familiar with computers may also have been important. A group with less prior computing experience might have been less positive. Participants’ responses to the intervention will be further illuminated by our qualitative data, which will be reported elsewhere.

Results on the outcome measures were mixed. The prediction that intervention would benefit functional communication, as assessed by the CADL-2, was upheld. Evidence of gain was produced by all analyses. The first mixed ANOVA analysis produced a significant interaction, indicating that the group that received intervention between week 1 and week 7 improved, whereas the waitlist control group did not. The follow up within group analyses were also significant. Encouragingly, these showed that the waitlist control group also improved once therapy was received. The CADL-2 is a standardised measure of context bound, everyday communication. For example, test items relate to attending a medical appointment, dealing with a receptionist and using the telephone. The assessment therefore examines skills that are very close to the situated goals that were identified and practised by most of our participants.

Other predicted changes were not achieved. We hypothesised that communication confidence would benefit, as a result of the diverse communication activities undertaken in EVA Park, and because of the supportive nature of the virtual environment. However, the Communication Confidence Rating Scale in Aphasia failed to show a difference between those who had and had not received intervention. The null result arose mainly because the waitlist control group improved on the measure even before they received intervention. This may suggest that simply being enrolled onto the study, and undergoing the assessments, enhanced confidence. It may also point to difficulties with the stability of the measure, which is still under development [61, 62]. Assessing communicative confidence in aphasia is challenging; and, to date, the tool used in our study is the only measure available. This factor may be captured more effectively by qualitative methods. Our interview data included many comments about the positive impact of EVA Park on feelings of confidence.

The opportunities to engage with others in EVA Park were predicted to reduce feelings of isolation. However, there was no significant change on the Friendship Scale. This may be due to the limited duration of therapy or because feelings of isolation are resistant to change. It may also be because virtual contacts are perceived as different from those in the real world.

Although not strongly predicted, possible changes in word production, conversation and narrative were explored. Word production, as assessed by a verbal fluency task, did improve over time, but changes could not be attributed to intervention. Rather all participants, including those in the waitlist control group, improved on each testing occasion. There was a tendency for scores to improve most on the categories that related to the content of EVA Park, particularly over the intervention periods. However, this trend was not significant. There is good evidence that word production skills in aphasia can be improved by speech and language therapy [64]. However, gains typically follow highly targeted interventions, for example involving repeated cued naming of a set of pictures [65], or semantic feature analysis [66, 67]. Such interventions were not delivered in EVA Park.

Conversation was assessed by the POWERS measure, with no significant findings. This is perhaps disappointing, given that conversation was a key activity in EVA Park. However, improving conversation in aphasia is difficult, even following conventional face to face therapy [68]; and when there is success in this area it typically follows the training of conversational partners [69]. Mere conversation practice, as experienced in EVA Park, may be insufficient to address this complex dimension of communication.

Narrative scores were unchanged by EVA Park intervention. Previous studies have reported gains in aphasic narrative production [70, 71]. However, these followed intervention that targeted specific language skills, such as sentence formulation. It seems that the more general language stimulation offered in EVA Park may not affect narrative skills.

The final analyses explored whether the amount of time spent logged into EVA Park affected change. Evidence for this was minimal. Most correlations between the gain scores on the outcome measures and individual log times were not significant. There were two exceptions. One, for the percentage of content words in conversation, was negative, suggesting a rogue result. The other was for the measure of communicative confidence (CCRSA). This is again difficult to interpret, given that the CCRSA had not demonstrated a therapy induced change. It is possible that CCRSA scores affected usage, rather than the other way round. In other words, those who felt more confident may have been more willing to make independent use of EVA Park. The CADL-2 results, which had responded to intervention, were subject to an ANCOVA analysis, with time logged into EVA Park as the covariate. This was not significant. There may be two main reasons for these largely negative findings. The usage data only reflected the amount of time spent in EVA Park, not what happened during that time. Finer grained data might be more informative. For example, the amount of interactive (rather than solitary) experience in EVA Park may correlate with gain. The second reason relates to this. Opportunities for independent language practice in EVA Park are currently few. Therefore those with high log-in times, over and above their supported sessions, were probably mainly engaged in non-language activities, such as exploring the visual features of the island. It is not surprising that this did not correlate with change.

Before considering the implications of this study, some limitations need to be acknowledged. Group assignment was not fully randomised, and although there were no significant differences between the groups at recruitment it is striking that scores on all measures were lower for the waitlist control group. There was also quite a large discrepancy in the time post stroke, albeit not significantly so. The sample size was small, raising concerns about both type 1 and type 2 errors. The sample may also have been atypical of stroke, particularly with respect to age. The average age of people experiencing a first stroke in the UK is over 70 [72], whereas the mean age of our sample was 57.8. On some of our measures (verbal fluency and CCRSA), scores were unstable even before intervention was received. Given the chronic nature of our sample this is unlikely to reflect recovery. Rather, it would point to problems with test/re-test reliability. Choosing appropriate outcome measures for aphasia therapy is challenging. Tools designed for the general population are often unsuitable, because of their language demands; while specialist measures may be unavailable or still under development. There is also a lack of consensus in the aphasia research community about the best outcome measures to use [73].

A further limitation of the study relates to the nature of the comparison that was conducted, which was EVA Park intervention vs no intervention. This allows no conclusions to be drawn about the relative merits of therapy delivered in virtual reality compared to ‘conventional’ face to face therapy. We were not attempting to address the latter question, partly because of the preliminary nature of this research, i.e. this was the first exploration of multi user virtual reality in aphasia therapy. Finding an appropriate comparator intervention would also be challenging. There is no gold standard aphasia therapy [7]. Rather, various approaches have been documented, which typically target specific language functions through exercises and drills e.g. [74, 75, 76, 77, 78]. Such activities were not delivered in EVA Park during this study. Therefore, any comparator would differ on more than simply the virtual component, making results difficult to interpret.

Despite these limitations some promising findings emerged from this study. It showed that communication intervention can be delivered on a bespoke virtual reality platform to people with aphasia. Good compliance and the lack of attrition showed that this intervention was accessible and acceptable to participants. In terms of outcomes this study showed that five weeks of supported language stimulation delivered in EVA Park brought about significant gains on a test of functional communication. This is an important outcome, although it should be replicated in a larger study to increase our confidence that it is a true effect. Functional communication has been cited as the primary goal of aphasia therapy [7], since it reflects the ability to communicate in real world settings. The opportunity to locate intervention in simulations of such settings is a key contribution of EVA Park.

A number of questions could be addressed in future research. It would be beneficial to trial the intervention with a larger, older and perhaps less computer literate sample, to test its broader application with the stroke population. Testing with participants who are in an earlier stage of recovery might also provide a better indication of treatment effectiveness. Further development of the platform would also be beneficial, particularly to increase the opportunities for independent language practice. Such developments would make it possible to deliver and evaluate formal therapy tasks in EVA Park, targeting specific aspects of language. The potential of EVA Park to deliver a range of support services might also be tested, such as social groups, peer support and befriending. This is the first application of multi user virtual reality in aphasia rehabilitation. The full potential now needs to be explored.

## Supporting Information

S1 File Study protocol. (DOCX)

S2 File Study data. (SAV)

S3 File Boxplots of dependent variables. (DOCX)

S1 Table TREND Checklist. (DOC)
