# Supplementary material for: Evaluating the Benefits of Aphasia Intervention Delivered in Virtual Reality: Results of a Quasi-Randomised Study
Source: PLoS One. 2016 Aug 12;11(8):e0160381. doi: 10.1371/journal.pone.0160381 (PMC4982664; doi:10.1371/journal.pone.0160381)
Supplement: S1 File — (DOCX) [file pone.0160381.s001.docx]

**Evaluating a virtual communication environment for people with aphasia**

**S1 File: Study Protocol**

**Background**

Aphasia occurs in about a third of stroke survivors (Engelter et al, 2006). There are typically long term negative consequences for well being (Cruice et al, 2011), including social isolation (Parr, 2007). Although aphasia responds to speech and language therapy (Bhogal et al, 2003), changes in the functional or everyday uses of language are difficult to achieve (Marshall, 2005). It has been argued that such changes require treatments that are embedded within ‘authentic’ social contexts (Simmons-Mackie, 2008). Exploiting virtual reality technologies may be one means of achieving this.

New computer technologies have been used to address a range of aphasic symptoms (e.g. Archibald et al, 2009; McCall et al, 2009; Mortley et al, 2004). But uses of virtual reality are rare. The few exceptions (e.g. Horvath et al 2009; Cherney et al, 2008; Lee et al, 2009) have not exploited interactive platforms. These provide a virtual world in which online personae, or avatars, interact within 3D simulated environments, using either speech or text. Environments mirror all aspects of life, with buildings, parks, cities and virtual events, such as parties. There have been numerous health related uses of virtual reality, e.g. in training medical students (Weicha et al, 2010), delivering patient information (Beard et al, 2009) and therapy (Gorini et al, 2008; Rose et al, 2005). People with physical disabilities have also reacted positively to its empowering opportunities (e.g. Cassidy, 2008; Jarmon et al, 2009). Yet, its potential for people with aphasia has not been tested. Here, it could be used to practise social uses of language within simulated scenarios, such as visiting the doctor. It might also provide a ‘safe’ environment that scaffolds language use and reduces feelings of social isolation.

**Research Questions**

This study aims to evaluate the benefits of a virtual environment for people with aphasia. The main questions are: Will access to the environment improve participants’ communication skills (assessed by a test of everyday functional communication)? Will access improve communicative confidence and reduce social isolation (assessed through a confidence rating scale, a measure of friendship and a measure of social networks)? Will there be secondary language gains (assessed through measures of verbal fluency, word finding in conversation and narrative)? Will effects be maintained? What are participants’ views about the virtual environment and its accessibility? The study will also inform us about the challenges presented to people with aphasia by this type of technology, and how these can be overcome.

**Methodology**

*The Virtual Environment*

A protected island will be created for the project within an existing virtual reality platform. This will offer a number of ‘real life’ scenarios, such as a shop, a doctor’s surgery, a restaurant and a bar. People in the island will interact with speech, rather than text. This Virtual Environment (VE) will be created by a technological researcher, a Human Computer Interaction (HCI) researcher and a Speech and Language Therapy (SLT) researcher. The team will work with 4 consultants with aphasia (not study participants), who will provide user opinion e.g. about the selection of scenarios and how they should be presented. Consultants will also advise us on navigation options, so that users can easily access the VE. Consultation sessions will be recorded, to illuminate factors that facilitate and inhibit access to the technology.

*Participants*

20 people with moderate aphasia will be recruited to trial the VE. They will be pre-morbid users of English, at least 4 months post stroke and will score within the following ranges on screening assessments (all from the Comprehensive Aphasia Test, Swinburn et al 2004): Spoken picture naming (at least 20% correct); Word to picture matching (>70% correct); Sentence to picture matching (>chance). These criteria will ensure that participants have sufficient language to participate in the virtual environment. For the same reason, they will have no uncorrected visual impairment and no hearing loss above 40 Db.

Participants will be recruited from community groups for people with stroke and aphasia. They will give written consent. Information and consent materials will be designed to be accessible to people with aphasia.

*Design*

The study will be both experimental and qualitative. The experimental aspect will employ a quasi-randomised controlled design, comparing outcomes across groups that have immediate and delayed access to the VE. The qualitative aspect will employ interview and observation techniques.

Following screening, participants will undergo baseline assessments (time point 1). Those in the immediate condition will then have 5 weeks access to the VE. The assessments will be repeated after this period (time point 2) and again 5 weeks later (time point 3). Those in the delayed condition will receive no intervention for 5 weeks, after which the assessments will be repeated (time point 2). They will then have 5 weeks access to the VE, followed by a third administration of the assessments (time point 3).

Participants will be recruited in four cohorts. Two cohorts will be assigned to the immediate condition and two to the delayed condition. Assignment of the cohorts will be random and will be determined before recruitment begins.

*Intervention*

Intervention will be provided in the VE. Access to the VE will be restricted to the project team, the study participants and to a team of support workers. These workers will be recruited and trained by the project team and will interact with participants while they are in the VE; i.e. they will play roles, such as a shop assistant or house guest. Support workers will have prior experience of working with aphasic people, e.g. because they are SLT graduates. The VE will be live four times over the course of the study, so each period will run 5 participants. In live periods the participants will have daily scheduled sessions with the support workers. Live periods will each last 5 weeks.

During live periods participants will access the VE from home using a lap top provided by the project. Where necessary the project will also fund an internet connection. Participants’ use of the VE will be supported by the SLT researcher. S/he will visit participants three times at home, to introduce the technology and trouble shoot problems, and will participate in VE sessions, to provide virtual support.

Each participant will receive 5 weeks of intervention. They will be allocated to a support worker who will help them to set communication goals and to work towards those goals using activities in the VE. Anticipated activities will include role plays, conversation and discussion groups.

*Measures*

Screening and profiling assessments will comprise The Comprehensive Aphasia Test (Swinburn et al, 2004), pure tone audiometry, and a questionnaire about prior computer use (Roper, unpublished).

The following outcome measures will be administered at each time point by an assessing speech and language therapist:

- Functional Communication: Participants will be assessed on CADL-2 (Communication Activities of Daily Living, Holland et al, 1999). This is a standardised assessment of everyday language use for people with aphasia, which is based on specific scenarios, such as going to the doctor. It has demonstrated good inter-scorer and test-retest reliability (Holland et al, 1999).
- Communicative Confidence: The Communication Confidence Rating Scale for Aphasia (CCRSA, Babbitt & Cherney, 2010) will be used. This asks ten questions relating to different aspects of communication, such as ‘How confident do you feel about your ability to talk with people?’ Confidence is rated on a 0 – 100 scale, where 0 is ‘not confident’ and 100 is ‘very confident’. The measure is still under development, so psychometric testing is ongoing (Babbitt et al, 2011; Cherney et al, 2011). It is the only published confidence measure designed to be used with people who have aphasia.
- Social Isolation: The Friendship Scale (Hawthorne, 2006) will be administered at each time point. This is a simple, 6 item measure with good reliability and discriminant validity. It explores feelings of loneliness and social connection.
- The Social Networks Analysis (Antonucci & Akiyama, 1987) will be used to explore the number, range and frequency of participants’ social contacts. This is a simple and reliable measure that has been used successfully with aphasic people (Cruice et al, 2006).
- Word retrieval: This will be assessed using a verbal fluency task employing categories that are and are not related to the virtual environment.
- Spoken narrative: Participants will be asked to retell a familiar story. They will be videoed and their production will be transcribed, analysed and scored using standard methodology (Berndt et al, 2000).
- Conversation: Participants will be filmed in conversation with an unfamiliar partner at each time point. Volunteer students of speech and language therapy will be trained to act as partners and will be randomly assigned to participants over the 3 time points. A ten minute sample will be extracted from each conversation, using consistent sampling criteria, and analysed using the POWERS procedure. This provides indices of word retrieval in conversation, such as the number of turns containing substantive vocabulary. The procedure has demonstrated good inter-rater reliability and sensitivity to therapy induced change (Herbert et al 2008, Best et al, in press).

Qualitative interviews will be conducted and filmed with each participant at time point 1 and at the end of the study. These will be semi-structured and will explore participants’ experiences of everyday communication, their social activities, their use of social and virtual technologies, and barriers and facilitators, both in the real and virtual world. For example, the interviews will explore whether participants have accessed Second Life beyond the protected VE of the project, and their responses to this access. The interviews will be conducted and analysed by the assessing therapist.

HCI Assessment: Participants will be visited twice while they have access to the VE by the Human Computer Interaction researcher. The first visit will occur in week 1 and the second in week 5. They will be observed during a virtual session, so that ease of access to the technology can be explored, and their views elicited about it.

Participants’ use of the VE will be electronically monitored, e.g. with respect to the number of hours spent. A sample of virtual sessions will also be recorded and analysed. This will offer insights into participants’ use of language in the environment, and whether this changes over time.

*Data Analysis*

Quantitative test data (CADL, CCRSA, Friendship Scale, Social Networks Analysis, verbal fluency, narrative, and conversation) will be analysed with mixed analyses of variance. Benefit from the environment will be demonstrated by an interaction between group and time, with the immediate group improving significantly more at time point 2 than the delayed group. Data from time point 3 will show whether the delayed group now demonstrates a benefit from the environment and whether the previous benefits of the immediate group have been maintained. Interview data will be coded and analysed for emergent themes (Ritchie & Spencer, 1994), exploring participant experiences and the perceived value of the VE. A sample of transcripts will be coded independently by two members of the research team to check reliability.

*Service User Involvement*

We will employ four consultants with aphasia in the first year of the project. They will trial different technological options and give us feedback about them, both verbally and through demonstration. Thus we will sample user opinion while we are developing the VE, in a detailed and ‘aphasia friendly’ way.

**Plans to Manage and Deliver the Project**

The project team will comprise an HCI researcher (.2 FTE), a technological researcher (.5 FTE in year 1), a speech and language therapy (SLT) researcher (.4 FTE in year 1 rising to .6 in years 2 & 3), and an assessing therapist/SLT (.4 FTE in years 2 & 3).

The VE will be developed in Year 1 by the technological researcher, working in partnership with the HCI and SLT researcher. An iterative developmental cycle will be employed, with technological prototypes being trialed with the aphasic consultants, and modified in response to their feedback. The support workers will be recruited and trained at the end of year 1 by the HCI and SLT researchers. Recruitment and screening of aphasic participants by the SLT researcher will begin.

In year 2 and 3 the twenty participants will complete the study (10 in each year). Experimental measures and qualitative interviews will be administered by the assessing therapist, who will also recruit and train the SLT students for the conversational assessment. Access to the environment will be supported by the SLT researcher, and will be evaluated by the HCI researcher. Support workers will be managed by the project PI, with day to day input from the SLT and HCI researchers. Data analysis will be conducted by the assessing therapist, HCI researcher and by the applicant team. All applicants will contribute to dissemination of findings, e.g. via publications and conference presentations. The full project team will meet monthly throughout the duration of the project.

**Ethical Issues**

There are 2 key participant issues; the first relates to obtaining informed consent from people who have compromised understanding of language. We will address this by compiling aphasia friendly materials for the project, including an accessible information sheet and consent form. The research team has extensive experience in designing and using such materials. Information will be delivered by a qualified speech therapist, who is skilled in communicating with people who have aphasia. In addition a minimum level of language skill will be required to participate.

The second relates to safeguarding participants whilst using the virtual environment. We have addressed this by making the virtual communication environment a protected private space that is only accessible by the project team and participants. Project staff will have enhanced CRB checks.

Several measures will ensure the confidentiality of data. All paper records will be stored in an anonymised system – i.e. with identifying details separated from research scores. Identifying participant details will not be disclosed to anyone working outside of the project. Most video recordings will only be seen by the research team and scorers. The scores will be University staff or students who are educated about clinical confidentiality. Confidentiality issues will be reiterated prior to their involvement in our research. Only with participant consent will any recordings be viewed by people outside the project team, e.g. for teaching or conference purposes. Video recordings will be stored securely on a password-protected computer and/or external hard drive. All portable records (e.g. files, discs, hard drives) will be kept in a lockable cabinet within City university premises

Researchers will adopt safe lone worker practices, e.g. when visiting participants at home. These will include keeping an electronic diary of visits and texting on arrival/departure.

**Predicted outcome of the research**

The research will inform us about the potential benefits of a virtual communication environment for people with aphasia. It will indicate whether access to the VE enhances communication, and changes feelings of social connectedness. For example, participants may report that they feel more in touch with others and that they are undertaking more social activities. Our qualitative methodology will also help us to identify barriers to change, e.g. arising from problems of access to mainstream social networking technologies. The project will create an aphasia friendly virtual world. If findings are positive, we will seek to make this available, on a long term basis, to the aphasia community. Such a resource might be of particular interest to stroke survivors in remote areas, who are not able to access local groups, and to young stroke survivors.

**Impact**

There will be impacts for users, service providers and relevant academic groups. For users and service providers the project will create a virtual environment that could supplement face to face services, particularly for young stroke survivors or people living in remote areas. It will also generate insights into how to support people with aphasia in accessing technology. Relevant academic groups include rehabilitation and HCI researchers. Here findings may stimulate follow up investigations, e.g. into interventions that combine face to face and virtual therapies. Insights into the nature of aphasia will be generated by our test and VE language samples. Our dissemination activities will target users and service providers, as well as academics. So, in addition to academic publications, we will present our findings to stroke groups, clinical conferences and on user websites, such as Aphasia Now. We will maintain a project website that is accessible to stroke survivors.

**References**

Antonucci, T. & Akiyama, H. (1987) Social networks in adult life and a preliminary examination of the convoy model. Journal of Gerontology, 42, 519 – 527.

Archibald, L., Orange, J. and Jamieson, D. (2009) Implementation of computer based language therapy in aphasia. Therapeutic Advances in Neurological Disorders, 2, 299-311.

Babbitt E, and Cherney L. (2010) Communication confidence in persons with aphasia. Topics in Stroke Rehabilitation, 17, 197–206

Babbitt E., Heinemann A., Semik P., and Cherney, (2011) L. Psychometric properties of the Communication Confidence Rating Scale for Aphasia (CCRSA): Phase 2. Aphasiology, 25, 6–7, 727–735

Beard L., A Wilson K., Morra D., and Keelan J (2009) Survey of Health-Related Activities on Second Life. J Med Internet Res. 11(2): e17. Published online 2009 May 22. doi: 10.2196/jmir.1192.

Berndt, R., Wayland, S., Rochon, E., Saffran, E. and Schwartz, M. (2000) Quantitative Production Analysis (QPA) Hove: Psychology Press

Best, W; Grassly, J., Greenwood, A., Herbert, R., Hickin, J. and Howard, D. (2011) A controlled study of changes in conversation following aphasia therapy for anomia. Disability and Rehabilitation , 33 (3) pp. 229-242.

Bhogal, S., Teasell, R., and Speechley, M. (2003) Intensity of Aphasia Therapy, Impact on Recovery. Stroke, 34, 987 – 993.

Cassidy, M. (2008) Flying with disability in Second Life. Eureka Street, 18, 1, 22-24.

Cherney, L., Halper, A., Holland, A. and Cole, R. (2008) Computerized Script Training for Aphasia: Preliminary Results. American Journal of Speech Language Pathology, 17, 1, 19 – 34.

Cherney L., Babbitt E., Semik P., and Heinemann A. (2011) Psychometric Properties of the Communication Confidence Rating Scale for Aphasia (CCRSA): Phase 1, Topics in Stroke Rehabilitation, 18, 352-360.

Cruice M., Worrall L. and Hickson, L. (2006) Quantifying aphasic people’s social lives. Aphasiology 20, 12, 1210

Cruice, M., Worrall, L. and Hickson, L. (2011). Reporting on psychological well-being of older adults with chronic aphasia in the context of unaffected peers. Disability and Rehabilitation, 33(3), pp. 219-228.

Engelter, S., Gostynski, M., Papa, S., Frei, M., Born, C., Ajdacic-Gross, V., Gutzwiller, F., and Lyrer, P. (2006) Epidemiology of Aphasia Attributable to First Ischemic Stroke. Stroke, 37, 1379-1384.

Gorini, A., Gaggioli A., Vigna,C., and Riva G. (2008) A Second Life for eHealth: Prospects for the Use of 3-D Virtual Worlds in Clinical Psychology J Med Internet Res. 10(3): e21. Published online 2008 August 5. doi: 10.2196/jmir.1029.

Hawthorne, G. (2006) Measuring social isolation in older adults: Development and initial validation of the Friendship Scale. Social Indicators Research, 77, 521 – 548.

Herbert, R., Hickin, J., Howard, D., Osborne, F. and Best, W. (2008). Do picture naming tests provide a valid assessment of everyday functional lexical retrieval? Aphasiology. 22/2, 184-203.

Holland,A., Frattali, C. and Fromm, D. (1999) Communication Activities of Daily Living-2. Austen TX: Pro-Ed.

Horváth, M., Dániel, C., Stark, J., and Lanyi, C. (2009) Virtual Reality House for Rehabilitation of Aphasic Clients. Transactions on Edutainment, Lecture notes in computer science, Volume 5940.

Jarmon L, Lim K.Y.T., and Carpenter B. (2009) Pedagogy , Education and Innovation in 3-D Virtual Worlds ” Innovation 2 (1) April 2009.

Lee, J., Kaye, R. and Cherney, L. (2009) Conversational script performance in adults with non fluent aphasia: Treatment intensity and aphasia severity. Aphasiology, 23, 7/8, 885 – 897.

Marshall J (2005) Can speech and language therapy with aphasic people affect activity and participation levels? A review of the literature. In P.W. Halligan & D.T. Wade (Eds) The effectiveness of rehabilitation for cognitive deficits. Oxford: Oxford University Press.

McCall, D., Virata, T., Linebarger, M., and Berndt, R.S. (2009) Integrating technology and targeted treatment to improve narrative production in aphasia: A case study. Aphasiology, 23:4, 438-461.

Mortley, J., Wade, J., and Enderby, P. (2004) Superhighway to promoting a client-therapist partnership? Using the internet to deliver word-retrieval computer therapy. Aphasiology, 18, 193 – 211.

Parr, S. (2007) Living with severe aphasia: Tracking social exclusion, Aphasiology, 21, 1, 98 – 123.

Ritchie, J. and Spencer, L. (1994) Qualitative data analysis for applied policy research. In A. Bryman & R. Burgess (eds) Analysing Qualitative Data. London: Routledge, 173 – 194.

Simmons-Mackie, N. (2008) Social approaches to aphasia intervention. In R. Chapey (ed) Language Intervention Strategies In Aphasia and Related Neurogenic Communication Disorders. Baltimore: Lippincott Williams and Wilkins.

Rose, F, Brooks, B. and Rizzo, A.. (2005) Virtual reality in brain damage rehabilitation: Review. Cyber Psychology and Behaviour, 8, 3, 241 – 262.

Swinburn, K., Porter, G. and Howard, D. (2004) Comprehensive Aphasia Test. Hove: Psychology Press.

Weicha, J., Heyden, R., Sternthal, E., and Merialdi, M. (2010) Learning in a Virtual World: Experience with Using Second Life for Medical Education. Journal of Medical Internet Research, 12, 1.
