# Supplementary material for: Evaluating the Benefits of Aphasia Intervention Delivered in Virtual Reality: Results of a Quasi-Randomised Study
Source: PLoS One. 2016 Aug 12;11(8):e0160381. doi: 10.1371/journal.pone.0160381 (PMC4982664; doi:10.1371/journal.pone.0160381)
Supplement: S3 File — (DOCX) [file pone.0160381.s003.docx]

**Evaluating the benefits of aphasia intervention delivered in virtual reality:**

**Results of a quasi-randomised study**

**Supplementary material: S3 File**

This supplement provides box plots of dependent variables’ scores across time for the immediate and waitlist control groups. The box plots show the median as a dark line; the box represents the 25-75 centiles, i.e. the interquartile range; and the lines the full range of scores.

**Figure A: Boxplot of CADL scores across time for immediate (n=10) and waitlist control (n=10) groups**


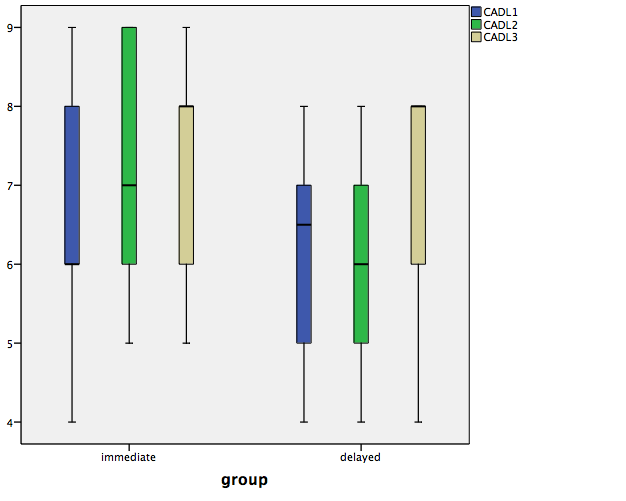


CADL: Communication Activities of Daily Living test scores

1: Week 1 (pre-therapy for immediate treatment group and pre-therapy 1 for waitlist control group)

2: Week 7 (post-therapy for immediate treatment group and pre-therapy 2 for waitlist control group)

3: Week 13 (maintenance for immediate treatment group and post-therapy for waitlist control group)

**Figure B: Boxplot of Verbal Fluency scores across time for immediate (n=10) and waitlist control (n=10) groups**


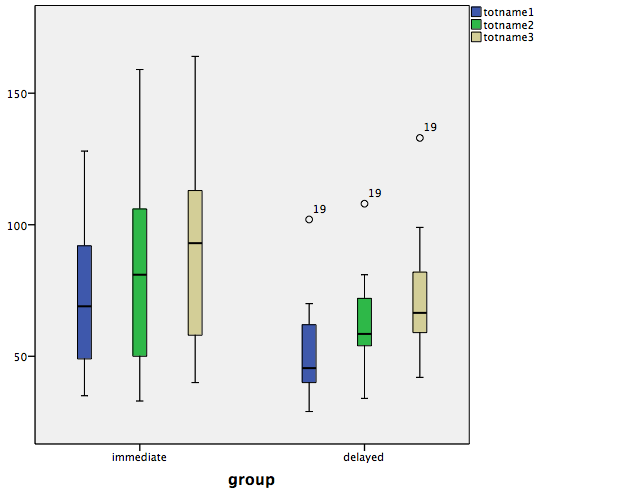


totname: Verbal Fluency scores

1: Week 1 (pre-therapy for immediate treatment group and pre-therapy 1 for waitlist control group)

2: Week 7 (post-therapy for immediate treatment group and pre-therapy 2 for waitlist control group)

3: Week 13 (maintenance for immediate treatment group and post-therapy for waitlist control group)

**Figure C: Boxplot of Conversation % content words scores across time for immediate (n=10) and waitlist control (n=10) groups**


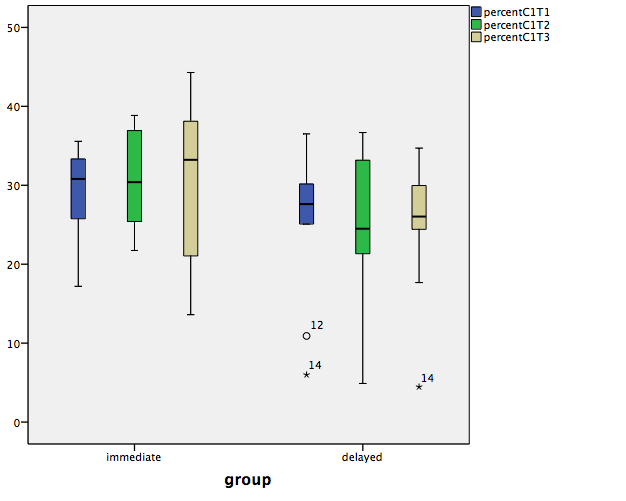


percentC1: Conversation % of content words scores

T1: Week 1 (pre-therapy for immediate treatment group and pre-therapy 1 for waitlist control group)

T2: Week 7 (post-therapy for immediate treatment group and pre-therapy 2 for waitlist control group)

T3: Week 13 (maintenance for immediate treatment group and post-therapy for waitlist control group)

**Figure D: Boxplot of Conversation content words per turn scores across time for immediate (n=10) and waitlist control (n=10) groups**


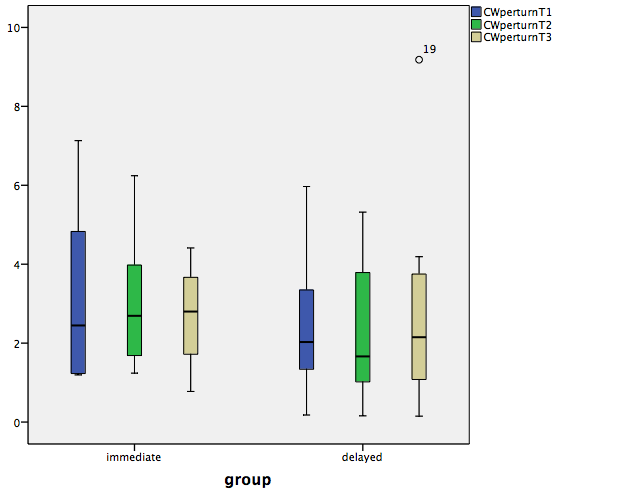


CWperturn: Conversation content words per turn scores

T1: Week 1 (pre-therapy for immediate treatment group and pre-therapy 1 for waitlist control group)

T2: Week 7 (post-therapy for immediate treatment group and pre-therapy 2 for waitlist control group)

T3: Week 13 (maintenance for immediate treatment group and post-therapy for waitlist control group)

**Figure E: Boxplot of Narrative Words per minute scores across time for immediate (n=10) and waitlist control (n=9) groups**


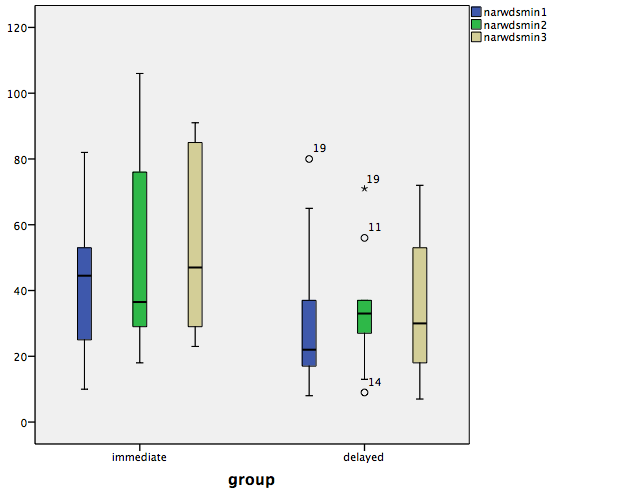


narwdsmin: Narrative words per minute scores

1: Week 1 (pre-therapy for immediate treatment group and pre-therapy 1 for waitlist control group)

2: Week 7 (post-therapy for immediate treatment group and pre-therapy 2 for waitlist control group)

3: Week 13 (maintenance for immediate treatment group and post-therapy for waitlist control group)

**Figure F: Boxplot of Narrative Sentences scores across time for immediate (n=10) and waitlist control (n=9) groups**


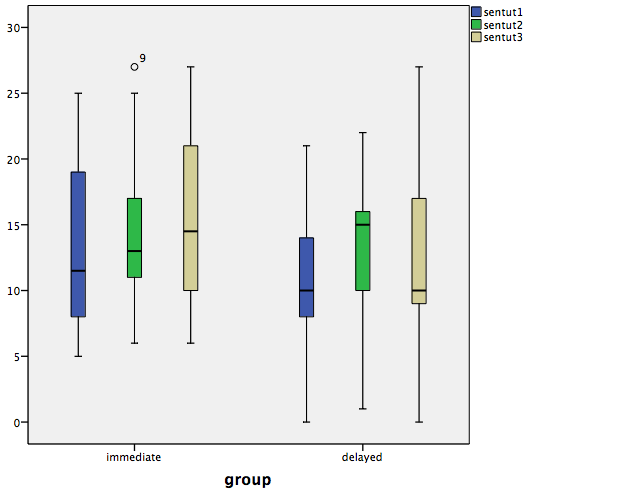


sentut: Narrative sentences scores

1: Week 1 (pre-therapy for immediate treatment group and pre-therapy 1 for waitlist control group)

2: Week 7 (post-therapy for immediate treatment group and pre-therapy 2 for waitlist control group)

3: Week 13 (maintenance for immediate treatment group and post-therapy for waitlist control group)

**Figure G: Boxplot of CCRSA scores across time for immediate (n=10) and waitlist control (n=10) groups**


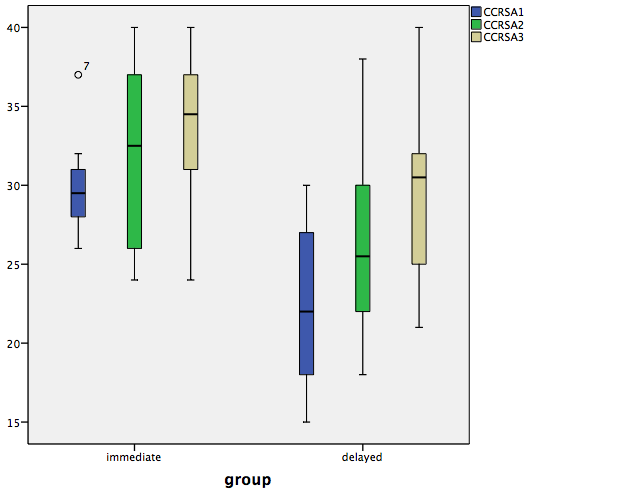


CCRSA: Communication Confidence Rating Scale for Aphasia scores

1: Week 1 (pre-therapy for immediate treatment group and pre-therapy 1 for waitlist control group)

2: Week 7 (post-therapy for immediate treatment group and pre-therapy 2 for waitlist control group)

3: Week 13 (maintenance for immediate treatment group and post-therapy for waitlist control group)

**Figure H: Boxplot of Friendship Scale scores across time for immediate (n=10) and waitlist control (n=10) groups**


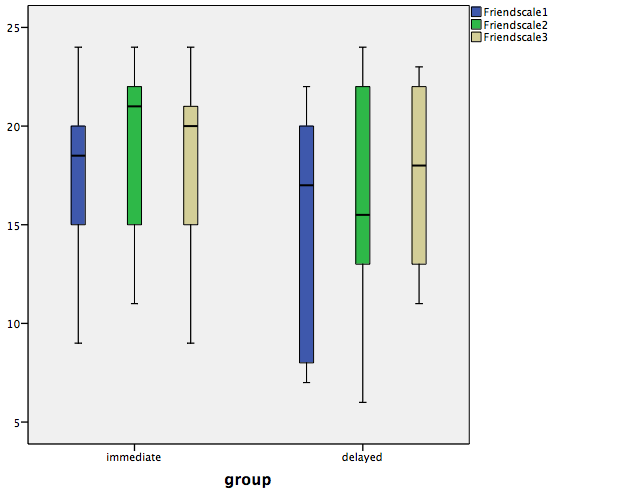


Friendscale: Friendship Scale scores

1: Week 1 (pre-therapy for immediate treatment group and pre-therapy 1 for waitlist control group)

2: Week 7 (post-therapy for immediate treatment group and pre-therapy 2 for waitlist control group)

3: Week 13 (maintenance for immediate treatment group and post-therapy for waitlist control group)
